# Suicidality and aggressive behaviors in migrants: evidence from an observational study

**DOI:** 10.1192/j.eurpsy.2026.11637

**Published:** 2026-07-31

**Authors:** B. Della Rocca, S. Cipolla, M. Di Vincenzo, D. Giallanella, F. Ricci, C. Toni, M. Luciano, A. Ventriglio, A. Fiorillo

**Affiliations:** 1 Department of Psychiatry, University of Campania “Luigi Vanvitelli”, Naples; 2 Department of Clinical and Experimental Medicine, University of Foggia, Foggia, Italy

## Abstract

**Introduction:**

Migrants often face traumatic experiences, acculturation stress, and barriers to care, which may increase vulnerability to suicidality and aggressive behaviors. These behaviors reflect emotional dysregulation and represent a significant concern in psychiatric inpatient settings.

**Objectives:**

To investigate sociodemographic, cultural, and clinical correlates of suicidality and aggression among migrant psychiatric inpatients, focusing on univariate analyses.

**Methods:**

We retrospectively reviewed clinical records of 268 migrant patients admitted to a psychiatric unit in Italy (2004–2019). Self-harm (suicidal ideation/attempts) and aggressive behaviours were identified through clinical assessment. Sociodemographic and clinical characteristics were compared between groups using chi-square and t-tests.

**Results:**

Self-harm was reported in 22.4% of patients. Compared with others, these patients were less often male and more frequently experienced acculturation stress (63.3% vs. 48.6%). They scored higher on BPRS suicidality (3.9 vs. 2.3), guilt (2.5 vs. 1.8), and hostility (3.2 vs. 2.1). Patients without self-harm more often displayed psychotic-spectrum symptoms such as unusual thought content, bizarre behavior, and distractibility. Aggressive behaviours was observed in 22.5% of patients, which were were younger, less educated, and less likely to have a partner compared to those who did not present aggressive behaviours. Moreover, they more often faced precarious legal status (33.3% with residence permit vs. 57.1%) and lower Italian fluency. Clinically, they were more frequently involuntarily admitted (25.4% vs. 2.4%), had higher BPRS total scores (60.8 vs. 49.4), and showed marked hostility, motor hyperactivity, and suspiciousness. Their functioning was significantly lower (GAF 36.7 vs. 44.1). The overlap between suicidality and aggression was minimal (3.7%).

**Conclusions:**

Our findings suggest that suicidality in migrants is linked to acculturation stress and internalized symptoms, while aggression relates to social vulnerability factors such as poor integration, unstable legal status, and limited support. These risks are not rooted in ethnicity but reflect structural and social challenges. Strengthening integration pathways, legal stability, and culturally sensitive care may reduce both self-harm and aggression in migrant patients.

**Disclosure of Interest:**

None Declared

